# Intrapapillary hemorrhage with adjacent peripapillary subretinal hemorrhage of both eyes after COVID-19 infection: a case report

**DOI:** 10.1186/s12886-024-03368-y

**Published:** 2024-03-04

**Authors:** Yifan Wang, Hong Chen, Lifei Yuan, Yijia Fan, Yilei Liang, Haiyu Zhang, Ziyao Dang, Lifei Wang

**Affiliations:** 1 Department of Ophthalmology, Hebei Medical University, 050017 Shijiazhuag, Hebei China; 2 Hebei Eye Hospital, XingTai, Hebei China

**Keywords:** Intrapapillary hemorrhage with adjacent peripapillary subretinal hemorrhage, Intrapapillary hemorrhage, Optic disc, Subretinal hemorrhage, Myopia, COVID-19 infection

## Abstract

**Background:**

Intrapapillary hemorrhage with adjacent peripapillary subretinal hemorrhage is commonly observed in myopia with tilted optic disc. It presents with typical features on the fundus and follows a self-limiting course. However, due to its complex etiology, clinicians sometimes lack sufficient understanding of it which can easily lead to misdiagnosis or overtreatment. In this case report, we describe a rare case of intrapapillary hemorrhage with adjacent peripapillary subretinal hemorrhage in both eyes.

**Case presentation:**

An 18-year-old female who has no past medical history experienced sudden black shadow blocking of her right eye in the right eye for the past 2 days after a 5-day history of COVID-19 infection. The best corrected visual acuity is 0.5 in the right eye and 0.6 in the left eye. Optical coherence tomography (OCT) showed tilted optic discs in both eyes, bulged nasal optic discs, and the presence of strong reflective material under the parafoveal retina of the optic discs. Fundus fluorescein angiography (FFA) showed subretinal fluorescence occlusion above and nasolateral to the optic disc in the right eye, with hypofluorescence below the optic disc; the subretinal below the optic disc was obscured by vitreous hemorrhage; hypofluorescence was seen in the optic disc region of the left eye.COVID-19 antigen was positive. The patient was in the early stage of the third COVID-19 infection when the disease occurred. We speculate that it may be related to it. After 5 months of conservative treatment, the patient’s hemorrhage disappeared in both eyes and her best corrected visual acuity returned to normal.

**Conclusions:**

Intrapapillary hemorrhage with adjacent peripapillary subretinal hemorrhage usually occurs in myopia with tilted optic disc. In most patients, the cause of the bleeding is unknown, but it can gradually resolve under clinical observation or conservative treatment.

## Background

Intrapapillary hemorrhage with adjacent peripapillary subretinal hemorrhage is commonly observed in myopia with tilted optic disc. It presents with typical features on the fundus and follows a self-limiting course. However, due to its complex etiology, clinicians sometimes lack sufficient understanding of it which can easily lead to misdiagnosis or overtreatment. In this case report, we describe a rare case of intrapapillary hemorrhage with adjacent peripapillary subretinal hemorrhage in both.

## Case presentation

**Fig. 1:**
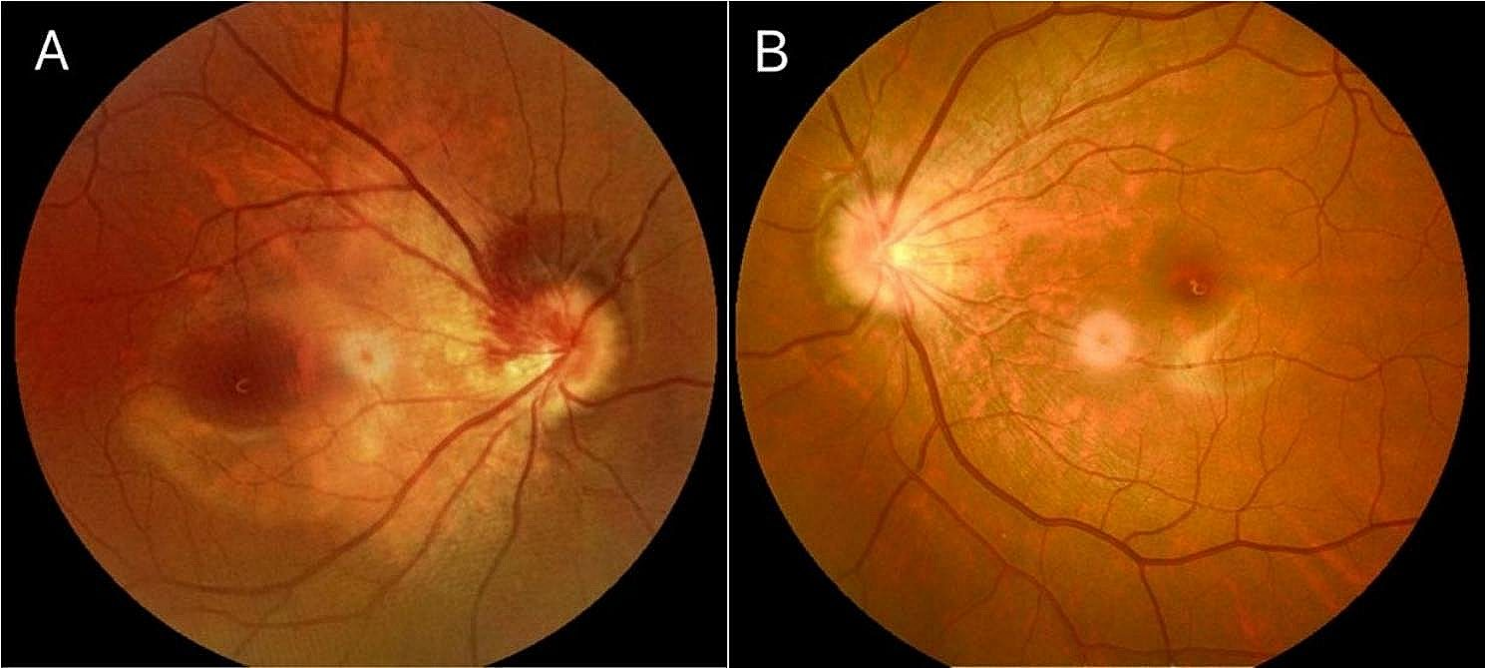
(**A**) Fundus photograph of the right eye showing a tilted optic disc, blood accumulation in the optic disc, and vitreous hemorrhage. (**B**) The left eye showing the blurred boundary of the optic disc

**Fig. 2:**
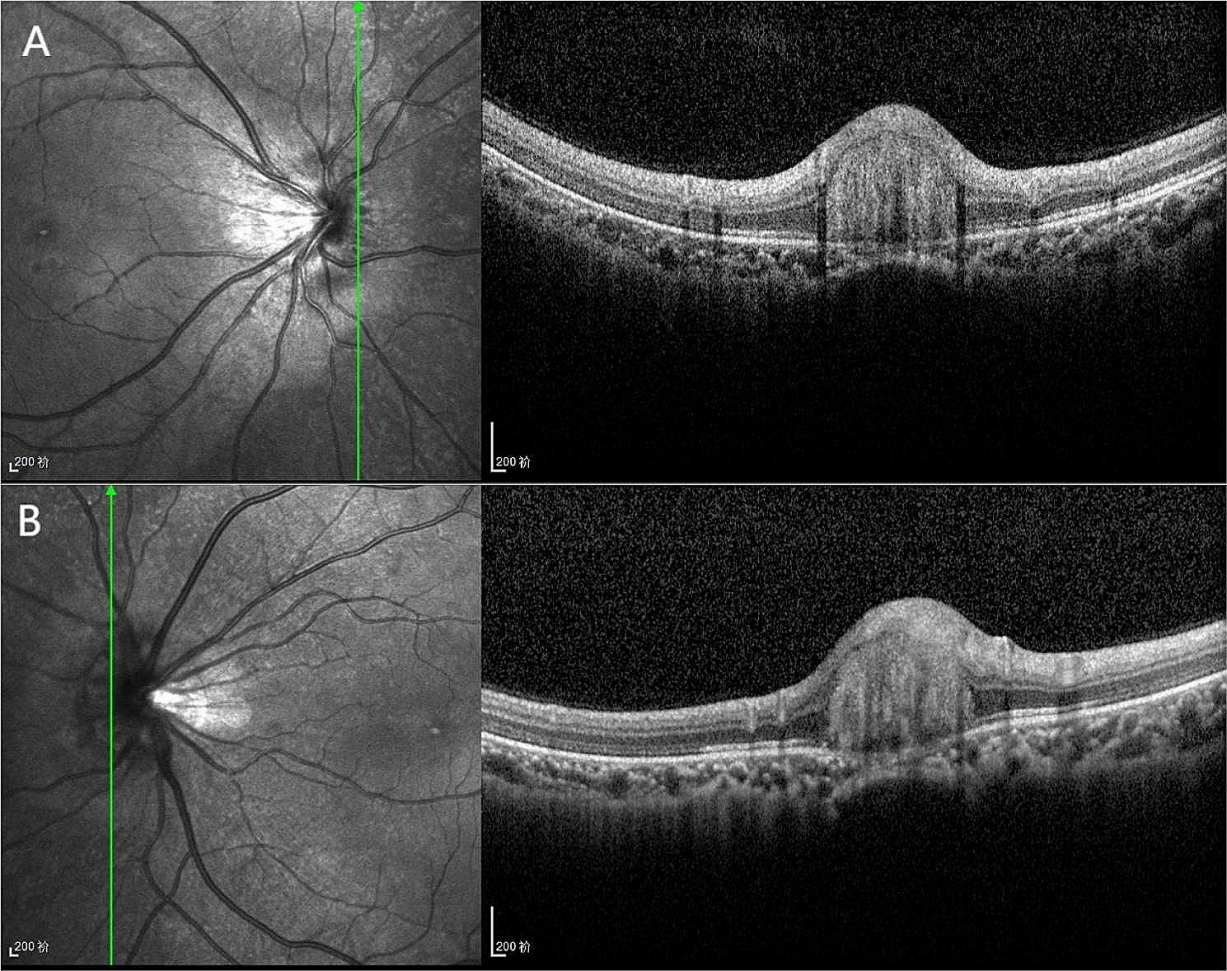
(**A**) Optical coherence tomography showing tilted optic discs, bulged nasal optic discs, and the presence of strong reflective material under the parafoveal retina of the optic discs in the right eye. (**B**) The left eye showing tilted optic discs, bulged nasal optic discs, and the presence of strong reflective material under the parafoveal retina of the optic

**Fig. 3:**
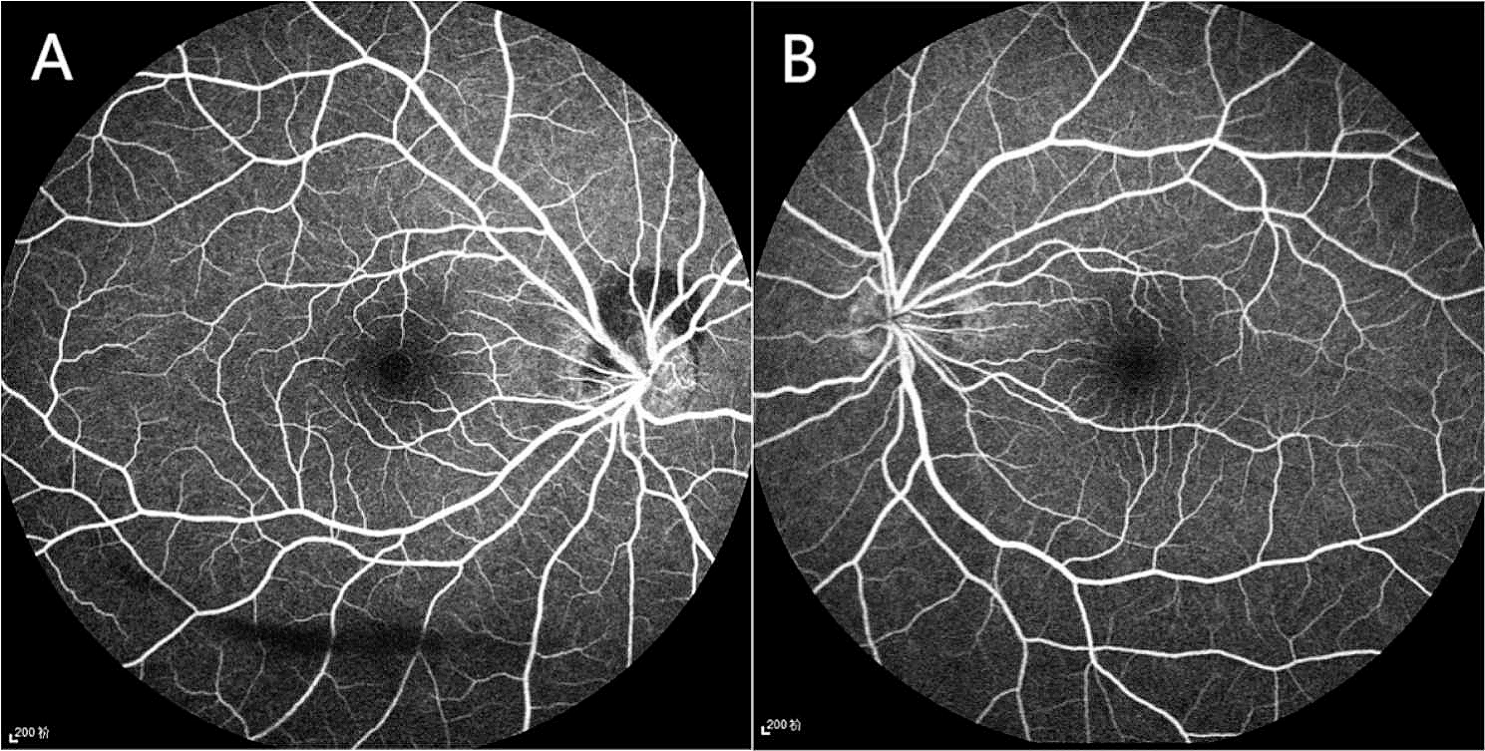
(**A**) The right eye showing subretinal fluorescence occlusion above and nasolateral to the optic disc, with hypofluorescence below the optic disc; the subretinal was obscured by vitreous hemorrhage. (**B**) The left eye showing hypofluorescence in the optic disc region

**Fig. 4:**
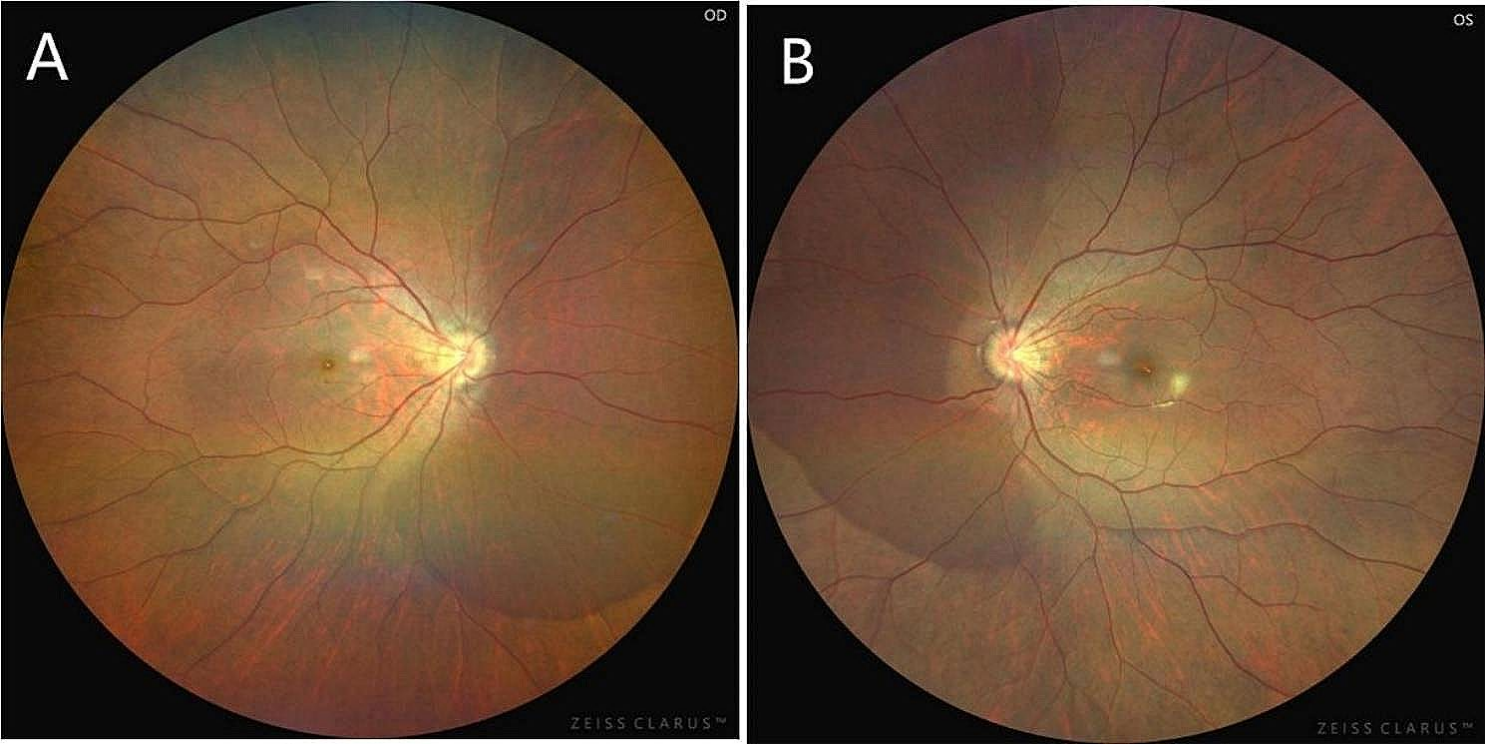
The hemorrhage before and below the optic disc had mostly resolved

The patient is an 18-year-old female who presented to our department on April 26, 2023,with a sudden occlusion of visual field in the right eye for the past 2 days after a 5-day history of COVID-19 infection. She was moderate myopic (right eye, -5.25 -1.00 × 164; left eye,-5.00 -0.50 × 175) and the COVID-19 antigen test was positive. No significant abnormalities were found in other examinations. She said she was in the early stages of her third COVID-19 infection. Ophthalmic examination: the BCVA in the right eye is 0.5,and in the left eye is 0.6. The intraocular pressure is 18 mmHg in both eyes. Anterior segment examination reveals no abnormalities. In the right eye, it showed a tilted optic disc, peripapillary and subretinal hemorrhage, and shallow “flame-shaped” hemorrhages in the superficial layers of the retina before the optic disc. In the left eye, there is a tilted optic disc with unclear disc boundaries. The retinal vessels appear normal without hemorrhages or exudates, a kidney-shaped spot is present on the nasal side of the retina and the macular centrails reflection is present in both eyes (Fig. 1). Optical coherence tomography (OCT) showed tilted optic discs in both eyes, bulged nasal optic discs, and the presence of strong reflective material under the parafoveal retina of the optic discs. This finding is more prominent in the right eye (Fig. 2). Fundus fluorescein angiography (FFA) showed masking of fluorescein in the nasal and superior regions of the optic disc during the arterial phase in the right eye. Gradual enhancement of fluorescence was observed in the inferior optic disc, with hypofluorescence in the late phase. The inferior retina of fluorescence was masked by vitreous hemorrhage (Fig. 3). The blood routine, coagulation function, and orbital magnetic resonance imaging (MRI) showed no abnormalities. The diagnosis is as follows: (1) Bilateral intrapapillary hemorrhage with adjacent peripapillary subretinal hemorrhage. (2) Bilateral refractive error. The patient was prescribed Zhixue Mingmu Granules(10 g, 3 times daily, orally) for hemostasis and improving vision and accepted anti-COVID-19 treatment at the same time. Close follow-up was conducted in the outpatient clinic. After 4 months, the hemorrhage had mostly resolved, and the visual acuity in the left eye had recovered to 1.0 but the in the right eye remained at 0.6. By the 5th month after the onset of symptoms, the patient’s visual acuity had fully recovered to 1.0 in both eyes (Fig. 4).

## Discussion and conclusions

The incidence and reports of this kind of characteristic disease of the optic disc and peripapillary subretinal hemorrhage are less,it is uniformly named intrapapillary hemorrhage with adjacent peripapillary subretinal hemorrhage(IHAPSH) [1]. The disease has the following clinical features: (1) adolescent onset; (2) Monocular onset; (3) Mild to moderate myopia; (4) titled optic disc; (5) acute onset of disease; (6) a good visual prognosis [2]. Most patients complain of floaters, blurring of vision, or deterioration of vision. Fundus examination shows titled optic disc, disc elevate of the nasal or superior disc margin, blurred boundary, and the small and crowded optic disk with small or missing cups. The dark-red or black-red adjacent peripapillary subretinal hemorrhage with a clear boundary around 1/3 − 2/3 of the disc margin is mostly located on the nasal side, even more on the nasal side. Or note the flame-shaped hemorrhage on the surface of the optic disc; A few cases are accompanied by vitreous hemorrhage which results in floaters, and in severe cases, the above 3 types of hemorrhage phenomena may coexist. When the hemorrhage is located within or below the optic disc, it usually causes a blurry sensation. However, when a large amount of bleeding enters the vitreous cavity, patients may experience a sense of visual obstruction. A preliminary diagnosis can be made by considering the patient’s general condition and fundus examination findings. Some researchers found a possible association between IHAPSH and COVID-19 vaccination, as well as inflammatory response. Kokame et al. [3] presented two cases in which IHAPSH occurred after Leber’s idiopathic stellate neuroretinitis, suggesting that inflammatory response could be an inducing factor for this condition. A previous study documented that 12 patients had retinal hemorrhage approximately two days after mRNA COVID-19 vaccination [4]. Tsuda et al. [5] reported a case of IHAPSH following mRNA COVID-19 vaccination. Unlike traditional vaccines that employ attenuated or inactivated pathogens, mRNA vaccines directly inject a small portion of mRNA into the body, which prompts the body’s cells to produce a partial or incomplete viral protein. This process triggers an immune response and establishes immune protection. Literature has reported cardiovascular events such as myocarditis and thrombosis after COVID-19 vaccination, and temporary changes in circulatory dynamics, including Vasospasm [6, 7]. Additionally, Honavar et al. [8] reported similar cases of optic disc hemorrhage after COVID-19 infection. Furthermore, there are many reports supporting the occurrence of various organ bleeding after COVID-19 infection, including cerebral hemorrhage, gastric bleeding, liver bleeding, kidney bleeding, non-menstrual abnormal uterine bleeding, and nasal bleeding. Although it’s common to observe hypercoagulable state and thromboembolism in COVID-19, bleeding can occur at any time during the infection, with a probability of 4.8-8% and the proportion of major bleeding is 3.5% [9, 10]. Qiu et al. [11] proposed that COVID-19 infection is a risk factor for hemorrhage. Although the mechanisms underlying bleeding in COVID-19 infection are not yet clear, several factors may play a role in it, such as vascular endothelial damage, endotheliitis, and hemodynamic abnormalities induced by immune system activation [9–11]. Li Xiaoxin pointed out that retinal vessels are part of intracranial vessels and have many similar pathological changes with intracranial vascular diseases. The choroidal blood flow is abundant and the flow velocity decreases after the systemic blood flows into the choroid, making it susceptible to deposition of pathogens and immune complexes which leads to disorders of the retina and choroid. In this case, IHAPSH occurred three days after COVID-19 infection corresponding to the timing of retinal hemorrhage reported after COVID-19 infection or COVID-19 vaccination, which mostly occurs around two days [4]. Our patient was infected with COVID-19 based on his abnormal optic disc structure, which is an important inducing factor, and the patient developed IHAPSH under the dual effect. Therefore, we speculate that there is a certain correlation between COVID-19 infection and the occurrence of IHAPSH in this case. The pathophysiological mechanisms of this disease are not fully clear and need to be differentiated from other conditions such as hemorrhage caused by optic disc drusen, hemorrhage caused by tractional retinal due to vitreous detachment, Valsalva retinopathy, glaucoma optic disc hemorrhage, Terson syndrome, and peripapillary intrachoroidalcavitation.In conclusion, the relevant reports of IHAPSH are rare, even more the bilateral onset. The pathophysiological mechanisms are still unclear. IHAPSH exhibits typical fundus manifestations and has a favorable self-limiting course.

## Data Availability

Data is provided within the manuscript or supplementary information files.

## Abbreviations

BCVA: Best corrected visual acuity
OCT: Optical coherence tomography
MRI: Magnetic resonance image
IHAPSH: Intrapapillary hemorrhage with adjacent peripapillary subretinal hemorrhage
